# *Helicobacter pylori* and Epstein–Barr Virus Infection in Gastric Diseases: Correlation with IL-10 and IL1RN Polymorphism

**DOI:** 10.1155/2019/1785132

**Published:** 2019-12-06

**Authors:** Fasciana Teresa, Nicola Serra, Giuseppina Capra, Chiara Mascarella, Cesare Gagliardi, Paola Di Carlo, Sara Cannella, Maria Rosa Simonte, Dario Lipari, Miriam Sciortino, Letizia Scola, Anna Giammanco

**Affiliations:** ^1^Department of Health Promotion, Mother and Child Care, Internal Medicine and Medical Specialties “G. D'Alessandro”, University of Palermo, Palermo, Italy; ^2^Department of Surgical, Oncological and Oral Sciences, Doctoral Program in Oncology and Experimental Surgery, University of Palermo, Palermo, Italy; ^3^Department of Public Health, University Federico II of Naples, Naples, Italy; ^4^Department of Biomedicine, Neuroscience and Advanced Diagnostics, University of Palermo, Palermo, Italy

## Abstract

**Introduction:**

*Helicobacter pylori* and Epstein–Barr virus (EBV) infection have recently been shown to be associated with gastric diseases. Polymorphisms in genes encoding cytokines such as interleukin 10 (IL-10) and interleukin 1 Receptor (IL-1RN) influence cytokine secretion levels and appear to contribute to the risk of developing gastroduodenal diseases. To our knowledge, this is the first preliminary study to address the association of coinfection with *H. pylori* and EBV and their correlation with genetic predisposition in the development of gastric diseases.

**Methods:**

Gastric biopsy samples of 96 patients with different gastric diseases were used.

**Results:**

Our results showed that the rate of coinfection was higher in patients with gastric cancer than in patients with normal gastric mucosa, active chronic gastritis, and MALT lymphoma. As regards the characterization of *H. pilory* strains, the polymorphism *s1m1i1* of *vacA* gene was more frequent in patients with MALT Lymphoma in comparison to others, while the polymorphism *s2m2i2* was most frequent in patients with normal gastric mucosa. In addition, patients who tested positive for the *cagA* gene were more frequently those affected with gastric cancer than those with inactive chronic gastritis. Similarly, the patients with *oipA* gene ON were more frequently those with gastric cancer than those with inactive chronic gastritis.

**Conclusion:**

According to our analysis, there was no correlation between coinfection and polymorphisms in genes encoding IL-10 and IL-1RN. We conclude that various factors can be involved in the development of gastric diseases.

## 1. Introduction

Gastrointestinal diseases and gastric cancer (GC) are among the most common pathologies worldwide [1]. In recent years, *Helicobacter pylori* and EBV infection seem to be strongly associated with the development of gastric diseases [2, 3].

*H. pylori*, a bacterium that colonizes the gastric mucosa of more than 50% of the world's population, is considered to be a group 1 carcinogen by the International Agency for Research on cancer [4].

Various virulence factors are involved in the development of gastric diseases in *H. pylori* positive patients; among these, the genes *cagA, vacA*, and *oipA* have an important role [5]. The cytotoxicity-associated gene A (*cagA*) is one of the most significant virulence factors of *H. pylori*. It is associated with the development of peptic ulcers and gastric cancer [6]. The *vacA* gene encoding for VacA protein is capable of inducing large host cell vacuoles in the cytoplasm of gastric cells [7], while the *oipA* (outer inflammatory protein) gene encodes one of the outer-membrane proteins that contribute to gastric inflammation, inducing IL-8 secretion by epithelial cells [8].

OipA expression is predicted to be regulated by a slipped strand mispairing system based on the number of CT dinucleotide repeats in the 5′ signal peptide coding region of the gene, with “ON” meaning *oipA* is functional and “OFF” nonfunctional. OipA functional status is involved in bacterial adherence to gastric epithelial cells and in mucosal inflammation.

Treatment failure in *H. pylori* infections is the current issue for physicians. There are many reasons for treatment failure. These can be grouped into microorganism-related factors, host-related factors, and treatment-related factors. *H. pylori* resistance to antibiotics is widely recognized as the chief reason for treatment failure.

*H. pylori* resistance to clarithromycin has been correlated to point mutations in the peptidyl transferase region of domain V of the *23S rRNA*. The most common mutation is an A-to-G transition at position 2143 (A2143G) and at position 2142 (A2143G).

EBV infection is present in more than 90% of the population. Upon infection, the virus rests latent in B lymphocytes throughout the person's lifetime [9].

Moreover, the association of EBV with gastric carcinoma was reported for a case of lymphoepithelial-like gastric carcinoma, but the mechanism used by the virus to determine the oncogenesis is still unknown [10].

It seems that EBV deregulation of the expression of immune response-related genes promotes marked intra or peritumoral immune cell infiltration [11]. The relationship of *H. pylori* with early inflammatory precancerous lesions is well known.

However, only a few studies have evaluated the participation of EBV infection in these lesions.

Some studies have found evidence that EBV infects epithelial cells of the atrophic gastric mucosa with a relatively low frequency [12], whereas other studies favour higher frequencies [13].

While both micro-organisms are responsible for the most common infections worldwide, a small percentage of infected patients will develop severe disease [14].

The outcome of the infection depends on the relationship between environment, host, and bacterial virulence factors [15, 16]. Recently, the polymorphism of proinflammatory cytokines such as IL1RN and anti-inflammatory cytokines such as IL-10 has been implicated in clarifying host factors in the development of gastric diseases [17].

In particular, IL1RN genotypes and low IL-10 production have been associated with an increased risk for severe gastric lesions in patients infected with both micro-organisms [18].

In this preliminary study, we analyze *H. pylori* and EBV coinfection and correlation with host genetic variability in gastric tissues from adult patients with different gastric diseases in Italy.

In this study, we considered four groups of patients: group with normal gastric mucosa (control group), group with active chronic gastritis, group gastric MALT (Mucosa-Associated Lymphoid Tissue) lymphoma, and group with gastric cancer. These groups were defined considering both premalignant disease and most common malignant disease correlated to micro-organisms such as MALT lymphoma and gastric cancer. The use of the groups was introduced to individualize the impact of each disease singularly and consequently to reduce possible statistical biases.

The investigation was deemed to be of interest as Sicily has one of the most complex mixed ethnic populations in Europe due to its geographical position.

## 2. Materials and Methods

### 2.1. Patients and Sample Collection

This study was performed on a sample of 96 patients, 34.38% males, and 65.62% females, aged 20–87 years, mean 58.27 y.o. and standard deviation (SD) 16.26 y.o.

### 2.2. We Considered Four Groups of 24 Consecutive Patients

- Group with normal gastric mucosa (Group NGM), composed of 33.33% males and 66.67% females, aged 22–71 years, mean 46.58 y.o. and standard deviation (SD) 14.74 y.o
- Group with active chronic gastritis (Group GCA) composed of 37.50% males and 62.50% females, aged 20–87 years, mean 49.04 y.o. and standard deviation (SD) 16.83 y.o
- Group with gastric cancer (Group KG), composed of 33.33% males and 66.67% females, aged 55–87 years, mean 69.88 y.o. and standard deviation (SD) 8.68 y.o
- Group with gastric MALT lymphoma (Group ML), composed of 33.33% males and 66.67% females, aged 57–80 years, mean 67.58 y.o. and standard deviation (SD) 6.24 y.o

Exclusion criteria were as follows: previous attempts to eradicate *H. pylori* and use of antibiotics or proton pump inhibitor within 2 weeks prior to endoscopy.

Each patient signed an informed consent form before undergoing endoscopy.

### 2.3. Sample Collection and Histological Analysis

Gastric biopsy samples were obtained from patients attending the endoscopy ward of the Endoscopy Services of the A.O.U.P. Paolo Giaccone (Palermo, Italy) and M. Raimondi Hospital, San Cataldo (Caltanissetta, Italy). Two biopsy samples were obtained from de the antrum and two from the gastric corpus. One was used for *H. pylori* culture and the second for histologic examination.

In patients suspected of having gastric cancer and MALT lymphoma, samples were taken directly from the gastric lesion.

One antrum sample and one corpus sample was fixed in formaline, paraffin-embedded, and stained with hematoxylin and eosin (HE). Inflammatory response was graded according to the Sydney System by a single experienced pathologist.

### 2.4. DNA Isolation from Gastric Biopsies, *H. pylori*, and EBV DNA Detection by PCR Methodology

DNA from the biopsy samples was extracted using a High Pure Template Preparation kit (Roche), in accordance with the manufacturer's instructions. The extracted DNA was stored at −20°C until use.

*H. pylori* infection was diagnosed when the *ureaseA* gene was detected with nested PCR, while the BAMHI-W fragment region of the EBV genome was used as the target to evaluate the presence of the virus, according to Di Carlo et al. [19] and Giardina et al. [20].

### 2.5. Detection of *cagA*, the EPIYA Motif, Typing of *vacA* Alleles, and Determining *oipA* Status

In order to detect the presence of the *cagA* gene and to analyse the EPIYA motif, the DNA from each strain was subjected to PCR, as described by Panayotopoulou et al. [21]. In determining the EPIYA motif, three different PCR products were obtained: EPIYA ABC (550 bp), EPIYA ABCC (650 bp), and EPIYA ABCCC (750 bp). The signal peptide (s) and midregion (m) of the vacA gene were amplified using the primers described by Atherton et al. [22]. Four different PCR products were obtained: *s1* (259 bp) and *s2* (286 bp) from the *s*-region and *m1* (290 bp) and *m2* (352 bp) from the *m*-region (Atherton et al., 1995). Two different PCR products were obtained from the *i*-region: *i1* (250 bp) and *i2* (260 bp) [23]. OipA status was determined in all strains by PCR and sequencing of the signal region of the *oipA* gene, according to Yamaoka et al. [24].

### 2.6. Determination of Point Mutations in the *H. pylori* 23S rRNA Gene

In order to detect mutations related to clarithromycin resistance in the *23S rRNA* gene and to establish the number of CT repeats present in *oipA,* the same primers used for the amplification gene were employed.

The resulting PCR products were subjected to gel electrophoresis in a 2% agarose gel, subsequently purified and concentrated for sequencing using Amicon Ultra 0.5 ml columns (Millipore). Purified amplicons were sequenced directly with the same forward and reverse primers used in the previous PCR, using an ABI PRISM BigDye Cycle Sequencing Ready Reaction kit (Applied Biosystems) according to the instructions supplied by the manufacturer. The analysis of products was performed using BioEdit v. 7.2.0 software according to Chiarini et al. [25] and Fasciana et al. [26].

### 2.7. Genotyping of Cytokine Polymorphisms

The IL-1RN exon 2 polymorphism was analyzed according to Rad et al. [27] Conditions were as follows: 95°C for 5 min, then 35 cycles of 95°C for 30 s, 50°C for 30 s, 72°C for 30 s, and finally 72°C for 5 min. The PCR products were analyzed by electrophoresis on a 2% agarose gel stained with ethidium bromide. Allele 1 (4 repeats) was 410 bp, allele 2 (2 repeats) was 240 bp, allele 3 (5 repeats) was 500 bp, allele 4 (3 repeats) was 325 bp, and allele 5 (6 repeats) was 595 bp in length, according to Forte et al. [28]. Amplification Refractory Mutation System (ARMS-PCR) was used to type 819C/T and 1082G/A IL-10 SNP, as described by Crivello et al. [30].

## 3. Statistical Analysis

Data are presented as number and percentage for categorical variables, and continuous data are expressed as mean ± standard deviation (SD), unless otherwise specified. The multiple comparison chi-square test was used to define significant differences among percentages: if chi-square test was positive (*p* value <0.05), then residual analysis with the *Z*-test to locate the highest or lowest significant presence was performed. To evaluate significant differences of means among groups, we performed the multicomparison ANOVA test. When the ANOVA test was positive (*p* value <0.05), pairwise comparisons were performed with Scheffé's test.

Univariate and multivariate linear correlation analysis was performed, where the test on Pearson's linear correlation coefficient *R* was performed with *t*-Student test, under null hypothesis of Pearson's linear correlation coefficient *R* = 0. In this step for dichotomies, we defined the experimental probability distribution for the variables *Gender*, *Hp, EBV*, and *Coinfected*, assigning them the following values:

- For Gender, male = 1 and female = 0
- Hp, presence = 1 and absence = 0
- EBV virus, presence = 1 and absence = 0
- Coinfected (patients with HP and EBV virus), yes = 1, and no = 0

For polymorphism variables *vacA*, IL-1RN, and IL-10, we defined, according to the frequency of the variable's modalities in our total sample data, the following scales, respectively:

- *s2m2i2* = 5, *s1m1i1* = 4, *s1m2i1* = 3, *s1m2i2* = 2, and *s1m1-m2i1* = 1 and polymorphism absence = 0
- 1/1 = 5, 1/2 = 4, 2/2 = 3, 1/3 = 2, and 4/4 = 1 and polymorphism absence = 0
- AA = 3, AG = 2, and GG = 1 and polymorphism absence = 0

For the gastric disease variables, we used the following scale:

- NGM = 1, GCA = 2, ML = 3, and KG = 4.

We considered all statistical tests with *p* value <0.05 to be significant. All data were analyzed with Matlab statistical toolbox version 2008 (MathWorks, Natick, MA, USA) for 32 bit Windows.

## 4. Results and Discussions

In this preliminary study, we evaluated the relationship between *H. pylori* and EBV infection, and cytokine polymorphism in gastric diseases in people living in the Mediterranean area [26, 30]. In our study, the positivity rate of *H. pylori* in patients with gastric cancer was higher than that found in patients with normal gastric mucosa, active chronic gastritis, and MALT lymphoma, namely, 50%, 45.83%, 41.67%, and 45.83%, respectively.

EBV was detected in 54.17% of the patients with gastric cancer. It was detected in smaller percentages in patients with normal gastric mucosa, active chronic gastritis, and MALT lymphoma, namely, 20.83%, 41.67%, and 37.50%, respectively. In any case, the rate of *H. pylori* in all patients analyzed was 45.83%, and the rate of EBV was 38.54%.

The rate of coinfection was higher in patients with gastric cancer than that in patients with normal gastric mucosa, active chronic gastritis, and MALT lymphoma, namely, 37.50%, 16.67%, 29.17%, and 25%, respectively. The *cagA* and *vacA* genes are commonly used as markers to characterize *H. pylori* virulence. Several epidemiological studies have reported geographical variations in the circulation of the virulence factors pertaining to the micro-organisms. In Sicily, the *cagA* gene is present in 52.27% of strains: in 97% of the strains, it is associated with the ABC EPIYA motif, and in most, it is associated with the presence of the *s1i1m1 vacA* allele.

In this study, the rate of *H. pylori* resistance to clarithromycin was high, occurring in 27% of the cases. The most frequent point mutation in the peptidyl transferase loop region of the *23S rRNA* gene was A2143G in 83% of resistant strains. These data confirm those reported by Fasciana et al. in a previous study [26].

Table 1 shows clinical information of the total patient sample, including age, gender, symptoms, and infection type, patients infected by *H. pylori*, by EBV or both (coinfected). In addition, we identify four groups according to symptom type: normal gastric mucosa (NGM), active chronic gastritis (GCA), gastric cancer (KG), and MALT lymphoma (ML).

In Table 2, we have summarized the results of our gene analysis. The table shows the percentages of polymorphism of *vacA* gene, polymorphism IL-1RN, and polymorphism IL-10 (−1082). In addition, in patients where the gene *vacA* was present, the percentage of those with gene *cagA* positive and *oipA* “ON” status is reported.

In Table 3, we have described in detail the four groups according to symptom type, reported in Table 1. For each group, we have reported age, gender, infection type (including patients infected by *H. pilory*, EBV, or both), and polymorphism *vacA* type. In patients where *vacA* was present, the percentage of those with gene *cagA* positive and *oipA* “ON” is indicated. Finally, in the last column, we report the results of univariate and multivariate analysis among groups.

In Table 3, we can observe a significant difference for age (*p* value <0.001); in particular, there was a significantly higher number of elderly patients in the gastric cancer and MALT lymphoma groups, in accordance with Ferlay et al. [1].

As regards the characterization of *H. pilory* strains, evaluated directly on gastric biopsies, the polymorphism *s1m1i1* of *vacA* gene was more frequent in Group ML (*p* value = 0.0169), while the polymorphism *s2m2i2* was most frequent in Group NGM (*p* value = 0.0359) and less frequent in Group KG (*p* value = 0.0276). In other words, the polymorphism *s1m1i1* was significantly associated with patients with MALT lymphoma, while the polymorphism *s2m2i2* was significantly associated with patients with inactive chronic gastritis.

In addition, the patients positive for *cagA* gene were more frequently those affected by gastric cancer (*p* value = 0.0198) and less frequently those with inactive chronic gastritis compared to patients with other diseases (*p* value = 0.0130). A similar situation was found for the *oipA* gene. Patients with *oipA* gene and “ON” status were more frequently those with gastric cancer (*p* value = 0.0198) and less frequently those with inactive chronic gastritis (*p* value = 0.0130).

Regarding number of CT repeats, the most prevalent in the “ON” frame status had six CT repeats. This was found to be the case in other studies [26, 30]. Among the out-frame status, “OFF” variants, the patterns with eight CT repeats were the most prevalent.

The evidence pointing to the *oipA* “ON” as a gastric cancer risk factor includes the ability of the bacterium carrying a functional *oipA* to attach to the gastric epithelial cells and induce inflammation, apoptosis, and a toxic effect on towards cultured gastric epithelial cell lines.

In Table 4, we can see the sequences of signal peptide-encoding region of *oipA*.

Analysis of the *23S rRNA* gene demonstrated that 32 (73%) patients were infected with *H. pylori*-susceptible strains and the remaining 12 (27%) with resistant strains (Table 2). The predominant point mutation observed among the 12 *H. pylori* resistant strains was A2143G in 10 cases (83 %) and A2142G in 2 cases (17%).

In Table 5, we report the frequency of polymorphism IL1RN and IL-10 loci for every group. In the last column, we report the results of univariate and multivariate analysis performed among the groups.

Table 5 shows that there was only one significant association between polymorphism IL-1RN and gastric disease. Particularly, patients with polymorphism IL-1RN type 1/2 were significantly less frequent in the ML Group (*p* value = 0.0248).

These results, in accordance with other reports, do not seem to attribute any particular role to proinflammatory or anti-inflammatory cytokine genotypes in gastric disease susceptibility [28].

Finally, in Table 6, we report the results of univariate and multivariate linear correlation analyses between polymorphisms IL-1RN and IL-10, with the independent variables age, gender, *H. pylori* infection, EBV infection, coinfection, and gastric disease. In this case, no correlations were found.

## 5. Conclusions

We can conclude that the various factors that can be involved in cancer development are very complex. A limitation of this study was the limited number of patients with the various gastric diseases. Therefore, further investigations are necessary to fully correlate the role of coinfection in gastric diseases in the Sicilian population. In addition, coinfection should be evaluated at an early age and in children with an increased risk of presenting more serious lesions later in life.

## Figures and Tables

**Table 1:** Clinical information: age, gender, symptoms, and infection type in the total patient sample.

Parameters	Sample data
Age	58.27 ± 16.26
Gender	
Male	34.38% (33/96)
Female	65.62% (63/96)
Symptoms	
NGM	25.00% (24/96)
GCA	25.00% (24/96)
ML	25.00% (24/96)
KG	25.00% (24/96)
Analysis with PCR	
Not infected	42.71% (41/96)
Infected by HP	45.83% (44/96)
Infected by EBV	38.54% (37/96)
Coinfected	27.08% (26/96)

NGM = normal gastric mucosa; GCA = active chronic gastritis; KG = gastric cancer; ML = MALT lymphoma; HP = *H. pylori*; coinfected = patients with *HP* and *EBV*.

**Table 2:** Gene analysis of our sample data.

Parameters	Sample data
Polymorphism of gene *vacA*	
*s1m1-m2i1*	2.27% (1/44)
*s1m2i2*	4.55% (2/44)
*s1m2i1*	6.82% (3/44)
*s1m1i1*	40.91% (18/44)
*s2m2i2*	45.45% (20/44)
Gene *cagA* (^†^)	
Negative	47.73% (21/44)
Positive	52.27% (23/44)
Gene *oipA* (^†^)	
“OFF”	47.73% (21/44)
“ON”	52.27% (23/44)
Polymorphism IL-1RN	
1/1	56.35% (54/96)
1/2	21.88% (21/96)
2/2	18.75 (18/96)
1/3	2.08% (2/96)
4/4	1.04% (1/96)
Polymorphism IL-10 (−1082)	
AA	47.92% (46/96)
AG	29.17% (28/96)
GG	22.92% (22/96)
*H. pylori* resistant	27.27% (12/44)
*H. pylori* sensible	72.73% (32/44)

HP = *H. pylori*; GCNA = inactive chronic gastritis; GCA = active chronic gastritis; KG = gastric cancer; ML = MALT lymphoma; ^†^cases where gene *vacA* was present.

**Table 3:** Characteristics of the groups: normal gastric mucosa (NGM), active chronic gastritis (GCA), gastric cancer (KG), and MALT lymphoma (ML). Results of univariate and multivariate analysis among groups.

Parameters	Group NGM	Group GCA	Group KG	Group ML	Univariate and multivariate analysis
Age	46.58 ± 14.74	49.04 ± 16.83	69.88 ± 8.68	67.58 ± 6.24	*p* < 0.001 (A) NGM < KG, *p* < 0.05^*∗*^ (Sh) NGM < ML, *p* < 0.05^*∗*^ (Sh) GCA < KG, *p* < 0.05^*∗*^ (Sh) GCA < ML, *p* < 0.05^*∗*^ (Sh) NGM < GCA, *p* > 0.05 (Sh) ML < KG, *p* > 0.05 (Sh)

Gender					
Male	33.33% (8/24)	37.50% (9/24)	33.33% (8/24)	33.33% (8/24)	*p*=0.987 (C)
Female	66.67% (16/24)	62.50% (15/24)	66.67% (16/24)	66.67% (16/24)	*p*=0.987 (C)

Analysis with PCR					
Not infected	50.00% (12/24)	45.83% (11/24)	33.33% (8/24)	41.67% (10/24)	*p*=0.987 (C)
Infected by HP	45.83% (11/24)	41.67% (10/24)	50.00% (12/24)	45.83% (11/24)	*p*=0.953 (C)
Infected by EBV	20.83% (5/24)	41.67% (10/24)	54.17% (13/24)	37.50% (9/24)	*p*=0.124 (C)
Coinfected	16.67% (4/24)	29.17% (7/24)	37.50% (9/24)	25.00% (6/24)	*p*=0.433 (C)

Polymorphism *vacA*					
*s1m1i1*	9.09% (1/11)	10.00% (1/10)	58.33% (7/12)	81.82% (9/11)	*p* < 0.0001 (C)^*∗*^ ML, *p*=0.0169 (Z)^*∗∗*^
*s1m2i1*	0.00% (0/11)	10.00% (1/10)	16.67% (2/12)	0.00% (0/11)	*p*=0.308 (C)
*s1m2i2*	9.09% (1/11)	0.00% (0/10)	8.33% (1/12)	0.00% (0/11)	*p*=0.589 (C)
*s2m2i2*	81.82% (9/11)	80.00% (8/10)	8.33% (1/12)	18.18% (2/11)	*p* < 0.0001 (C)^*∗*^ NGM, *p*=0.0359 (*Z*)^*∗∗*^ KG, *p*=0.0276 (Z)^*∗∗∗*^
*s1m1-m2i1*	0.00% (0/11)	0.00% (0/10)	8.33% (1/12)	0.00% (0/11)	*p*=0.435 (C)

Gene *cagA* (^†^)					
Negative	90.91% (10/11)	80.00% (8/10)	8.33% (1/12)	18.18% (2/11)	*p* < 0.0001 (C)^*∗*^ NGM, *p*=0.0130 (Z)^*∗∗*^ KG, *p*=0.0198 (Z)^*∗∗∗*^
Positive	9.09% (1/11)	20.00% (2/10)	91.67% (11/12)	81.82% (9/11)	*p* < 0.0001 (C)^*∗*^ KG, *p*=0.0198 (*Z*)^*∗∗*^ NGM, *p*=0.0130 (Z)^*∗∗∗*^

Gene *oipA* (^†^)					
“OFF”	90.91% (10/11)	80.00% (8/10)	8.33% (1/12)	18.18% (2/11)	*p* < 0.0001 (C)^*∗*^ NGM, *p*=0.0130 (Z)^*∗∗*^ KG, *p*=0.0198 (Z)^*∗∗∗*^
“ON”	9.09% (1/11)	20.00% (2/10)	91.67% (11/12)	81.82% (9/11)	*p* < 0.0001 (C)^*∗*^ KG, *p*=0.0198 (*Z*)^*∗∗*^ NGM, *p*=0.0130 (*Z*)^*∗∗∗*^

^*∗*^significant test; ^*∗∗*^significant most frequent; ^*∗∗∗*^significant less frequent; *A* = multicomparison ANOVA test; Sh = post hoc Scheffé's test for pairwise comparison; *C* = multicomparison chi-square test; *Z* = *Z*-test; HP = *H. pylori*; coinfected = patients with *HP* and *EBV*; NGM = normal gastric mucosa; GCA = active chronic gastritis; KG = gastric cancer; ML = MALT lymphoma; ^†^ = cases where gene *vacA* was present; *p* = *p* value.

**Table 4:** *oipA* CT repeat patterns.

Sequence of signal peptide-encoding region of *oipA*	No. CT	No. of strains
OFF status		
ATGAAAAAAGCTCTCTTA………CTCTCTCTCTCTCTCTCTCGTT	9	5
ATGAAAAAAGCCCTCTTACTAACTCTCTCTCTCTCTCTCGTTT	8	7
ATGAAAAAAGCTCTCTTGCTAACTCTCTCTCTCTCTCTCTCTCGTT	10	2
ATGAAAAAAGCTCTTTTA………CTCTCTCTCTCTCGTT	6	3
ATGAAAAAAGCTCTCTTACTAACTCTCTCTCTCTCTCGTT	7	2
ATGAAAAAGCTCTCTTA………CTCTCTCTCTCTCTCGTTCTGG	7	1
ATGAAAAAAGCCCTCTTA………CTCTCTCTTTCTCTCGTTTT	4 + 2	1
Total		**21**
ON status		
ATGAAAAAAGCTCTCTTACTAATTCTCTCTCTCTCGTT	5	4
ATGAAAAAAGCTCTCTTACTAACTCTCTCTCTCTCGTT	6	15
ATGAAAAAAGCCCTCTTA………CTCTCTCTCTCTCTCTCTCTCTCGT	11	2
ATGAAAAAAGCTCTCTTACTAACT CTCTCTCTCTCTCTCTCGTT	9	1
ATGAAAAAAGCTCTTTTACTCTCT CTCTCTCTCTCGTT	8	1
Total		**23**

**Table 5:** Frequency of the polymorphisms IL1RN and IL-10 loci. Results of univariate and multivariate analysis among groups.

Parameters	Group NGM	Group GCA	Group KG	Group ML	Univariate and multivariate analysis
Polymorphism IL-1RN					
1/1	50.00% (12/24)	62.50% (15/24)	41.67% (10/24)	70.83% (17/24)	0.179 (C)
1/2	29.17% (7/24)	25.00% (6/24)	33.33% (8/24)	0.00% (0/24)	0.0239 (C)^*∗*^ group ML^*∗∗∗*^, *p*=0.0248 (Z)
2/2	20.83% (5/24)	8.33% (2/24)	16.67% (4/24)	29.17% (7/24)	0.314 (C)
1/3	0.00% (0/24)	4.17% (1/24)	4.17% (1/24)	0.00% (0/24)	0.564 (C)
4/4	0.00% (0/24)	0.00% (0/24)	4.17% (1/24)	0.00% (0/24)	0.387 (C)
Polymorphism IL-10 (−1082)					
AA	41.67% (10/24)	45.83% (11/24)	54.17% (13/24)	50.00% (12/24)	0.841 (C)
AG	33.33% (8/24)	33.33% (8/24)	29.17% (7/24)	20.83% (5/24)	0.875 (C)
GG	25.00% (6/24)	20.83% (5/24)	16.67% (4/24)	29.17% (7/24)	0.768 (C)

^*∗*^Significant test; ^*∗∗*^significant most frequent; ^*∗∗∗*^significant less frequent; # = no localized significant modality with significant level <0.05; *C* = multicomparison chi-square test; *Z* = *Z*-test; HP = *H. pylori*; NGM = normal gastric mucosa; GCA = active chronic gastritis; KG = gastric cancer; ML = MALT lymphoma; *p* = *p* value.

**Table 6:** Results of univariate and multivariate linear correlation analysis between polymorphism of *vacA*, IL-1RN and IL-10 with age, gender, *H. pylori*, *EBV,* coinfected, and gastric disease.

Linear correlation analysis	Univariate analysis	Multivariate analysis
	*R* (*p* value)	Multiple linear correlation coefficient = 0.149
Polymorphism IL-1RN/age	−0.092 (0.371)	*R*_*partial*_ = −0.057; *p* value = 0.593
Polymorphism IL-1RN/gender	−0.038 (0.716)	*R*_*partial*_ = −0.013; *p* value = 0.905
Polymorphism IL-1RN/*H. pylori*	0.02 (0.846)	*R*_*partial*_ = −0.027; *p* value = 0.800
Polymorphism IL-1RN/EBV	−0.027 (0.794)	*R*_*partial*_ = 0.052; *p* value = 0.622
Polymorphism IL-1RN/coinfected	−0.015 (0.886)	*R*_*partial*_ = −0.011; *p* value = 0.917
Polymorphism IL-1RN/gastric disease	−0.097 (0.348)	*R*_*partial*_ = −0.107; *p* value = 0.314
	*R* (*p* value)	Multiple linear correlation coefficient = 0.215
Polymorphism IL-10/age	0.116 (0.259)	*R*_*partial*_ = 0.106; *p* value = 0.316
Polymorphism IL-10/gender	−0.142 (0.167)	*R*_*partial*_ = −0.125; *p* value = 0.237
Polymorphism IL-10/*H. pylori*	0.102 (0.322)	*R*_*partial*_ = −0.024; *p* value = 0.821
Polymorphism IL-10/EBV	0.081 (0.430)	*R*_*partial*_ = −0.054; *p* value = 0.619
Polymorphism IL-10/coinfected	0.101 (0.329)	*R*_*partial*_ = 0.062; *p* value = 0.560
Polymorphism IL-10/gastric disease	0.061 (0.552)	*R*_*partial*_ = 0.027; *p* value = 0.797

^*∗*^Significant test; *R* = Pearson's linear correlation coefficient; *R_partial_* = the partial correlation coefficient is the coefficient of correlation of the variable with the dependent variable, adjusted for the effect of the other variables in the mode; coinfected = patients with *H. pylori* and *EBV*.
